# ReStress mindset: An internet-delivered intervention that changes university students’ mindset about stress in the shadow of the COVID-19 pandemic

**DOI:** 10.3389/fpsyg.2022.1036564

**Published:** 2022-10-26

**Authors:** Konstantinos Karampas, Christos Pezirkianidis, Anastassios Stalikas

**Affiliations:** Laboratory of Positive Psychology, Department of Psychology, Panteion University of Social and Political Sciences, Athens, Greece

**Keywords:** stress, stress mindset, Acceptance and Commitment Therapy, internet-delivered interventions, university students stress, university students

## Abstract

The aim of this study is to evaluate “ReStress Mindset,” an internet-delivered intervention that combines the Stress Mindset Training Program (SMTP) with Acceptance and Commitment Therapy (ACT). To that end, the current study determined whether the pilot study’s findings on the intervention’s effectiveness on stress mindset and stress response among university students in the midst of the COVID-19 pandemic, remained for 3 months following the completion of “ReStress Mindset” intervention. Twenty-six participants were randomly assigned to an intervention (*N* = 12) and a control (*N* = 14) group. Participants in the intervention group attended 5 weekly sessions online, between the second and third waves of the COVID-19 pandemic. All participants completed self-report questionnaires (Stress Mindset Measure, Satisfaction With Life Scale, Depression Anxiety and Stress Scale-9, Perceived Stress Scale, Scale of Positive, and Negative Experience) before, at the end of the intervention and 3 months after the completion of the program. The “ReStress Mindset” intervention resulted in a statistically significant increase in “stress-is-enhancing” mindset (SIEM), life satisfaction, and self-efficacy against stress, as well as a statistically significant decrease in “stress-is-debilitating” mindset (SIDM), with these effects lasting 3 months after the program’s completion. The findings of this study suggest that university students could benefit from the “ReStress Mindset” intervention in order to cultivate and maintain a positive stress mindset and increase their life satisfaction and self-efficacy against stress, even during the COVID-19 pandemic or any other highly stressful period or crisis.

## Introduction

### Mental health in the shadow of the COVID-19 pandemic

On March 2020, World Health Organization [WHO] (2020) has declared the COVID-19 as a pandemic. Coronavirus disease (COVID-19), which is thought to have originated in Wuhan, China, in December 2019, has had a significant global impact on people’s mental health (Holmes et al., 2020). A systematic review and meta-analysis, during the early period of the COVID-19 crisis in China, found that stress was the most prevalent (48.1%) mental health consequence of the COVID-19 pandemic, followed by depression (26.9%) and anxiety (21.8%; Bareeqa et al., 2021). According to a review study, stress can have a direct and indirect impact on health through changes in health behaviors, which can result in a variety of negative mental and physical health effects, including an increased risk of infectious diseases like COVID-19 (O’Connor et al., 2021). Moreover, a systematic review and meta-analysis showed that 28.6% of people worldwide reported low levels of wellbeing during the COVID-19 outbreak (Nochaiwong et al., 2021).

University students’ mental health is a growing public health concern (Bewick et al., 2010; Gallagher, 2012). Many studies show that students experience higher levels of distress, depression, and anxiety than the general population at their age (Eisenberg et al., 2007; Bayram and Bilgel, 2008; Lipson et al., 2018). A systematic review and meta-analysis found that during the COVID-19 pandemic anxiety symptoms were more prevalent among university students compare to similar populations prior to the pandemic (Deng et al., 2021). Moreover, the detrimental effects of the COVID-19 outbreak have been associated to higher stress levels and lower self-rated health among university students (Zurlo et al., 2020; Ryerson, 2022). Also, it is well documented that during the COVID-19 pandemic, university students and young adults worldwide experienced severe lifestyle and mental health disruptions, with serious consequences for their wellbeing (Cao et al., 2020; Li et al., 2020; Liu et al., 2020; McGinty et al., 2020; Tang et al., 2020; Giuntella et al., 2021).

A study in China found that during the coronavirus disease outbreak, the prevalence rates of stress among 746,217 university students was 34.9%, while anxiety symptoms were 11.0% (Ma et al., 2020). In a survey study conducted in the United Kingdom during the COVID-19 pandemic, university students reported significant levels of anxiety and depression, with more than half reporting levels above clinical cutoffs, as well as low levels of resilience (Chen and Lucock, 2022). Also, a study conducted among Polish university students found that self-reported physical health and life satisfaction decreased significantly during three waves of the COVID-19 pandemic (Rogowska et al., 2021b). Moreover, university students who reported high levels of perceived stress were seven times more likely to indicate high anxiety disorder risk. Low life satisfaction was also found to be a predictor of higher anxiety levels (Rogowska et al., 2021b). In Greece, a survey study among 1,018 undergraduates during the COVID-19 quarantine, found significantly increased levels of depression, anxiety, stress, and negative affect, while life disruption and perceived threat of the disease were risk factors in all psychological distress measures (Kornilaki, 2022).

Furthermore, the COVID-19 pandemic has exacerbated known mental health risk factors and other health concerns among university students, while also compromising students’ academic outcomes and future prospects (Lederer et al., 2021). As a result of campus closures and strict social isolation and physical distancing measures enacted in response to the COVID-19 pandemic, tertiary education institutions have shifted to online learning platforms. This transition is likely to exacerbate academic and other stressors for university students, such as mounting financial problems, a lack of social relationships, housing and food insecurity, uncertainty about the future, insufficient computer skills, poor quality of online classes, online exams, academic performance, and future studies (Byrnes et al., 2020; Kapasia et al., 2020; Aristovnik et al., 2021; Lederer et al., 2021; Chen and Lucock, 2022).

Previous research has clearly demonstrated that university students are experiencing unprecedented disruption and uncertainty, demanding immediate action to mitigate the pandemic’s negative academic and psychosocial impact (Grubic et al., 2020; Kornilaki, 2022). To that end, developing and implementing adequate prevention and intervention programs at universities should be a top priority in the fight against the COVID-19 pandemic, both during and after this global crisis (Grubic et al., 2020; Cénat et al., 2021; Rogowska et al., 2021b; Kornilaki, 2022).

### The ReStress mindset intervention

There is mounting evidence that mindset influences not only intelligence (Dweck, 2008) and aging (Levy and Myers, 2004), but also the stress response (Crum et al., 2013). According to a growing body of research on mindset, changing individual’s mindset toward stress can have a significant impact on stress response (Crum et al., 2017). Stress mindset is conceptualized as one’s belief that stress itself has either enhancing or debilitating consequences for outcomes such as performance and productivity, health and wellbeing, learning and growth (Crum et al., 2013). Moreover, “stress-is-enhancing” (SIEM) and “stress-is-debilitating” (SIDM) mindsets can have a different impact on physiological and behavioral responses to stress (Crum et al., 2013). Furthermore, research suggests that stress mindset is related to perceived health and life satisfaction over and above the effects of amounts of stress, stress appraisals, and coping strategies (Crum et al., 2013).

Acceptance and Commitment Therapy (ACT; Hayes et al., 2011) is a transdiagnostic psychological intervention that aims to increase psychological flexibility through six core processes (cognitive defusion, acceptance, committed action, values, contact with the present moment, and self-as-context), all of which are important for improving mental health (Hayes et al., 2011). Psychological flexibility is defined as the ability to be mindful of experiences in the present moment, in an accepting and non-judgmental manner, as well as to take action guided by values in order to move toward who or what is important, even in the face of adversity (Hayes et al., 2006; Polk and Schoendorff, 2014; Levin et al., 2017). According to ACT, human suffering derives from psychological inflexibility, a core process in which behavior is rigidly guided by immediate psychological experiences rather than by the individual’s values or goals (Levin et al., 2017). Experiential avoidance is a major component of psychological inflexibility, in which individuals avoid negative internal experiences such as thoughts, emotions, bodily sensations, and memories (Kocovski et al., 2013). Previous research found that university students who received ACT intervention reported less stress, reduced anxiety and depressive symptoms, increased psychological flexibility and general mental health, as well as improved mindful acceptance (Levin et al., 2017; Grégoire et al., 2018).

A meta-analysis of internet-delivered interventions for mental health and wellbeing in university students found that these types of interventions can be effective and improve the functioning of university students (Harrer et al., 2019). The “ReStress Mindset” is an internet-delivered intervention program designed to change the stress mindset and stress response of Greek university students in the midst of the COVID-19 pandemic (Karampas et al., 2022). The modules of this program were formed by combining the Stress Mindset Training Program (SMTP; Crum, 2011; Crum et al., 2013) and the ACT matrix protocol (Polk and Schoendorff, 2014) into a unified psycho-educational intervention. The pilot study of the “ReStress Mindset” intervention demonstrated that combining both models in a single intervention benefited university students by providing them with a broader repertoire of strategies and tools to deal with their challenges (Karampas et al., 2022). Research also suggests that providing university students with a variety of coping strategies during this period of extreme uncertainty can help them deal with stress and improve their overall wellbeing (Rogowska et al., 2021a). Moreover, Hayes et al. (2019) argue that we must tailor interventions to specific people in specific contexts. In this regard, the “ReStress Mindset” intervention is a context-oriented program that acknowledges that psychological flexibility contains the ability to shift mindsets (Kashdan and Rottenberg, 2010). Furthermore, the “ReStress Mindset” intervention is built within the framework of evidence-based processes of change and intervention elements that move them forward (Hayes and Hofmann, 2018; Hofmann et al., 2021). Processes of change are defined as theory-based, dynamic, progressive, contextually bound, modifiable, and multilevel change mechanisms that occur in predictable, empirically established sequences oriented toward desirable outcomes (Hofmann and Hayes, 2019). The modules of the intervention are presented in Table 1.

**TABLE 1:** Modules of the “ReStress Mindset” intervention.

	Module 1 stress mindset	Module 2 rethinking stress toolkit	Module 3 ACT overview	Module 4 ACT matrix	Module 5 final
In module	The paradox of stress and the power of mindset	Three steps to a SIEM exercise	ACT overview and ranking your values exercise	ACT matrix and values form exercises	Review material
Homework	Mindfulness exercises	Mindfulness exercises	Mindfulness exercises	Mindfulness exercises	N/A

### The purpose of the present study

The aim of the present study is to evaluate “ReStress Mindset” intervention (Karampas et al., 2022). To that end, this study will determine whether the findings of the pilot study regarding the intervention’s effectiveness on stress mindset and stress response among university students in the midst of the COVID-19 pandemic (Karampas et al., 2022), will remain for 3 months following the completion of the intervention.

The pilot study implemented the “ReStress Mindset” intervention led university students to cultivate a positive stress mindset even during COVID-19 pandemic, although further studies are required to establish the stability of the results over longer periods of time (Karampas et al., 2022). The results of the pilot study also indicated an increase in the levels of life satisfaction and psychological resilience of the intervention group, while the levels of life satisfaction and psychological resilience of the control group decreased after the intervention (Karampas et al., 2022). Additionally, no statistically significant changes in positive and negative emotions and psychological symptoms, were observed following the intervention, possibly due to the severe psychological impact of the COVID-19 pandemic (Karampas et al., 2022).

Thus, the present study will focus on answering the following research questions: (1) Does the “ReStress Mindset” intervention result in higher levels of SIEM, 3 months following the completion of the intervention? (2) Does the “ReStress Mindset” intervention result in lower levels of SIDM, 3 months following the completion of the intervention? (3) Does the “ReStress Mindset” intervention lead to an increase in levels of positive emotions, self-efficacy against stress, and life satisfaction, 3 months following the completion of the intervention? (4) Does the “ReStress Mindset” intervention result in lower levels of depression, anxiety, perceived helplessness against stress, and negative emotions, 3 months following the completion of the intervention?

## Materials and methods

### Participants

The sample consisted of 26 undergraduate Psychology students (96.2% women), aging from 19 to 39 years old (*M*_*age*_ = 24.46). Participants were recruited from Panteion University of Social and Political Sciences in Greece by responding to an online invitation sent *via* newsletter email. The majority of the participants was unemployed (50%), while some participants were working part-time (30.8%) and others full-time (19.2%). Regarding the marital status of the participants, 43.2% of them were single, 46.2% were in a relationship, 7.7% were married, and 3.8% were divorced.

### Measures

#### Demographics

Participants were asked to report demographic information regarding their gender, age, marital and employment status.

(1) Stress Mindset Measure (SMM; Crum et al., 2013; Greek version: Karampas et al., 2020). In the SMM the participants are rating how strongly they agree or disagree with eight statements (e.g., the effects of stress are positive and should be utilized, the effects of stress are negative and should be avoided) on a 0 (strongly disagree) to 4 (strongly agree) scale. In the Greek study, two factors were identified instead of a single stress mindset factor representing two different mindsets on the effects of stress: either it is enhancing or debilitating.

(2) Satisfaction With Life Scale (SWLS; Diener et al., 1985; Greek version: Galanakis et al., 2017). The scale measures individual’s cognitive assessment of his/her life indicating satisfaction with life levels. The SWLS consists of five items rated on a 7-point Likert-type scale (1-Strongly disagree to 7-Strongly agree).

(3) Depression Anxiety Stress Scales-9 (DASS-9; Yusoff, 2013; Greek version: Kyriazos et al., 2018a). DASS-9 is an empirically derived version based on DASS-21 (Lovibond and Lovibond, 1995; Pezirkianidis et al., 2018). The DASS-9 measures three negative emotional states (a) depression, (b) anxiety, and (c) tension/stress. Respondents report the presence of 9 symptoms over the previous week using a Likert-type scale (0-Did not apply to me at all to 3-Applied to me very much or most of the time). The three subscales of the DASS-9 were each cumulatively scored between 0 and 9, with higher scores demonstrating poorer mental health.

(4) Perceived Stress Scale (PSS; Cohen et al., 1983; Greek version: Andreou et al., 2011). The PSS has been developed to measure general stress based on the conceptualization of stress as an appraisal of something threatening and that people cope with stress more or less effectively (Lazarus and Folkman, 1984). PSS items ask participants to reflect on the past month and includes questions such as “Have you been upset by something that happened unexpectedly?” and “Have you felt that you could not cope with all the things you had to do?” (Scale: 0 = never to 4 = very often).

(5) Scale of Positive and Negative Experience (SPANE; Diener et al., 2010; Greek version SPANE-8: Kyriazos et al., 2018b). SPANE-8 is a revised structure containing one general feeling per dimension instead of three in the original SPANE (Diener et al., 2010). This resulted in a briefer structure with four positive (Pleasant, Happy, Joyful, Contented) and four negative (Bad, Sad, Afraid, Angry) items. Items are scored on a Likert scale from 1 (very rarely or never) to 5 (very often or always).

### Procedure

All participants (*N* = 26) were informed on the purpose of the study and gave their informed consent online. Also, participants were randomly allocated in the intervention (*N* = 12) and control (*N* = 14) groups and completed an online battery of questionnaires, as described above, at the beginning (T1), at the end of the intervention (T2) and 3 months after the completion of the program (T3). The members of the control group were offered the opportunity to attend the intervention after the completion of the study. The intervention lasted 5 weeks and consisted of 2-h modules. The intervention took place in February and March 2021, between the second and third waves of the COVID-19 pandemic, with a follow-up in June 2021. The content for each module, as well as the assigned homework, are listed in Table 1.

### Statistical analysis

The data was analyzed using the Statistical Package for Social Sciences (SPSS), version 28. Firstly, we conducted preliminary analyses to test if the data is appropriate and, then, a two-way (2 × 3) repeated measures mixed design ANOVA was performed to answer the research questions.

## Results

### Preliminary analyses

For each of the study variables we computed the mean, standard deviation, and Cronbach’s alpha coefficient (see Table 2). The means show differences in the levels of specific variables between the three time-points. Also, the results based on the internal consistency coefficient indicate adequate reliability of all subscales except for the subscale of DASS-9 measuring stress and the subscale of SPANE-8 measuring negative emotions, whose internal consistency range from 0.60 to 0.76. Moreover, the Kolmogorov-Smirnov tests indicate that all variables follow a normal distribution in almost every time-point.

**TABLE 2:** Descriptive statistics, alpha levels, and normality coefficients of study variables for the three time-points (*N* = 26).

Variable	Time	*M*	*SD*	α	*K-S D*	*df*	*p*
SIEM	T1	1.57	0.95	0.91	0.182	26	0.026
	T2	2.28	0.88	0.85	0.102	26	0.200
	T3	2.25	0.96	0.89	0.154	26	0.116
SIDM	T1	2.58	0.84	0.78	0.154	26	0.112
	T2	1.77	0.89	0.82	0.195	26	0.012
	T3	1.84	0.95	0.86	0.154	26	0.113
Satisfaction with life	T1	4.85	1.14	0.86	0.105	26	0.200
	T2	5.21	0.87	0.78	0.104	26	0.200
	T3	5.04	1.01	0.84	0.137	26	0.200
Depression	T1	0.92	0.68	0.70	0.153	26	0.122
	T2	0.69	0.67	0.78	0.243	26	0.000
	T3	0.96	0.82	0.89	0.200	26	0.009
Anxiety	T1	0.88	0.72	0.70	0.168	26	0.057
	T2	0.76	0.55	0.70	0.131	26	0.200
	T3	0.78	0.69	0.80	0.182	26	0.027
Stress	T1	1.44	0.66	0.60	0.168	26	0.057
	T2	1.17	0.51	0.65	0.203	26	0.007
	T3	1.24	0.63	0.64	0.136	26	0.200
Perceived helplessness against stress	T1	2.18	0.56	0.70	0.142	26	0.187
	T2	2.10	0.57	0.78	0.123	26	0.200
	T3	2.09	0.63	0.80	0.148	26	0.146
Self-efficacy against stress	T1	2.32	0.67	0.86	0.130	26	0.200
	T2	2.59	0.57	0.81	0.085	26	0.200
	T3	2.50	0.65	0.86	0.129	26	0.200
Positive emotions	T1	3.32	0.76	0.90	0.168	26	0.057
	T2	3.58	0.63	0.73	0.105	26	0.200
	T3	3.49	0.72	0.87	0.144	26	0.174
Negative emotions	T1	2.69	0.80	0.62	0.133	26	0.200
	T2	2.41	0.69	0.65	0.142	26	0.188
	T3	2.60	0.89	0.76	0.115	26	0.200

### Two-way repeated measures mixed design ANOVA

A two-way (2 × 3) repeated measures mixed design ANOVA was conducted to test the main effect of time (before, after the end, and 3 months after the intervention) and the two-way interaction amongst time and condition (intervention and control) on the levels of SIEM, SIDM, depression, anxiety, stress, positive and negative emotions, life satisfaction, perceived helplessness, and self-efficacy against stress. The required assumptions were met.

The results indicated a statistically significant main effect of Time on SIEM [*F*_(2,48)_ = 21.627, *MSE* = 6.505, *p* < 0.001, η*_*p*_*^2^ = 0.47], SIDM [*F*_(2,48)_ = 23.196, *MSE* = 5.970, *p* < 0.001, η*_*p*_*^2^ = 0.49], experiencing of positive emotions [*F*_(2,48)_ = 3.205, *MSE* = 0.483, *p* = 0.049, η*_*p*_*^2^ = 0.12], self-efficacy against stress [*F*_(2,48)_ = 6.429, *MSE* = 0.539, *p* = 0.003, η*_*p*_*^2^ = 0.21], satisfaction with life [*F*_(2,48)_ = 5.922, *MSE* = 0.947, *p* = 0.005, η*_*p*_*^2^ = 0.20], depression [*F*_(2,48)_ = 3.345, *MSE* = 0.570, *p* = 0.044, η*_*p*_*^2^ = 0.12], and stress [*F*_(2,48)_ = 4.708, *MSE* = 0.616, *p* = 0.014, η*_*p*_*^2^ = 0.16]. More specifically, the findings indicate that the participants in the study report higher mean SIEM, positive emotions, self-efficacy against stress, and satisfaction with life levels and lower mean SIDM, depression, and stress levels after the intervention.

To shed more light in the aforementioned results, we tested for possible interactions. A statistically significant interaction between Time and Condition was demonstrated for SIEM [*F*_(2,48)_ = 13.118, *MSE* = 3.946, *p* < 0.001, η*_*p*_*^2^ = 0.35], SIDM [*F*_(2,48)_ = 18.040, *MSE* = 4.643, *p* < 0.001, η*_*p*_*^2^ = 0.43], positive emotions [*F*_(2,48)_ = 3.940, *MSE* = 0.593, *p* = 0.026, η*_*p*_*^2^ = 0.14], negative emotions [*F*_(2,48)_ = 3.362, *MSE* = 0.727, *p* = 0.043, η*_*p*_*^2^ = 0.12], perceived helplessness against stress [*F*_(2,48)_ = 3.629, *MSE* = 0.367, *p* = 0.034, η*_*p*_*^2^ = 0.13], self-efficacy against stress [*F*_(2,48)_ = 4.531, *MSE* = 0.380, *p* = 0.016, η*_*p*_*^2^ = 0.16], life satisfaction [*F*_(2,48)_ = 7.096, *MSE* = 1.135, *p* = 0.002, η*_*p*_*^2^ = 0.23], and depression [*F*_(2,48)_ = 4.282, *MSE* = 0.730, *p* = 0.019, η*_*p*_*^2^ = 0.15].

**FIGURE 1:**
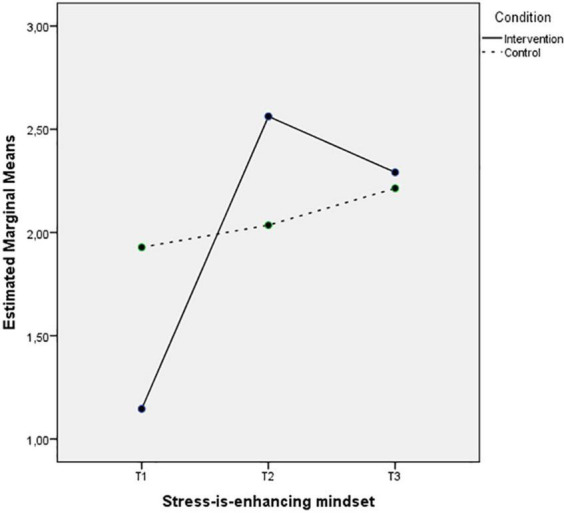
Mean of SIEM showing time effects for control and intervention groups.

**FIGURE 2:**
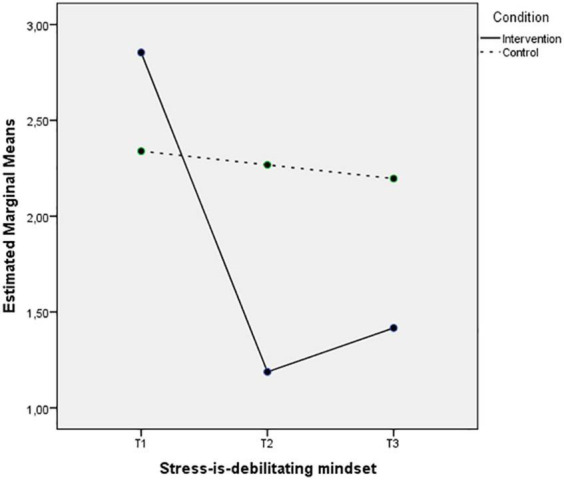
Mean of SIDM mindset showing time effects for control and intervention groups.

Figures 1, 2 depict the interaction between Time and Condition for SIEM and SIDM, respectively. The first figure depicts an increase on the intervention group levels of SIEM after the intervention that remains 3 months after and slight changes on the levels of the control group. The second figure shows a decrease of SIDM on the intervention group at the second time-point that remains in the follow up measurement, while the SIDM levels of the control group remain unchanged.

**FIGURE 3:**
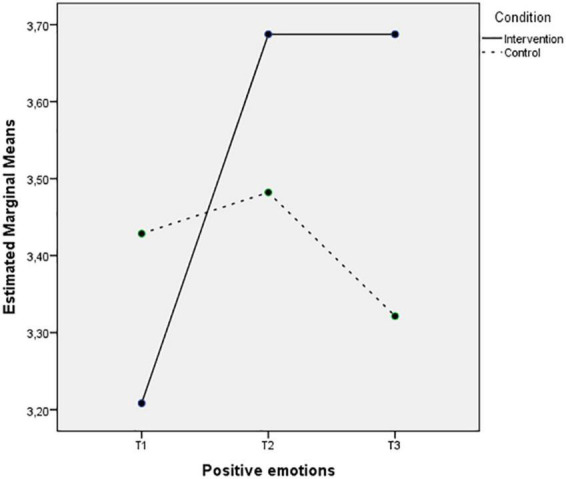
Mean of positive emotions showing time effects on the two conditions.

**FIGURE 4:**
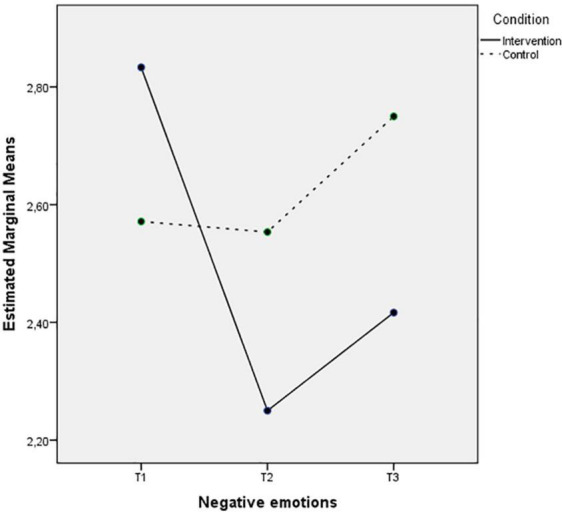
Mean of negative emotions showing time effects on the two conditions.

Figures 3, 4 depict almost the same pattern in the interaction between Time and Condition for the experiencing of positive and negative emotions. The intervention group levels of positive emotions increase, and the negative emotions decrease after the intervention and these levels remain 3 months after the intervention. However, the control group levels on both variables do not change in the second time-point, but the levels of positive emotions seem to decrease, and the negative emotions increase during Time 3.

**FIGURE 5:**
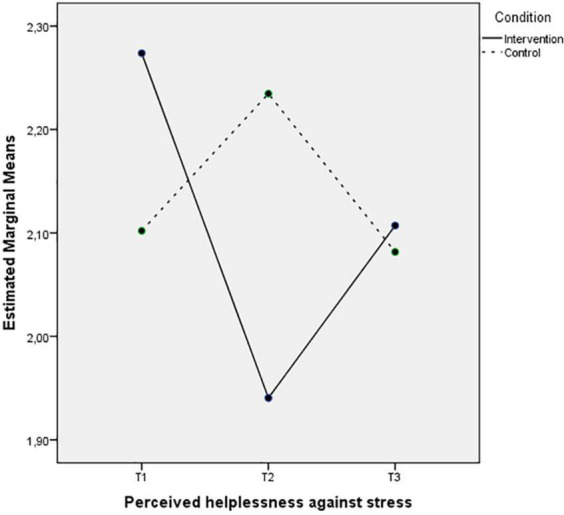
Mean of perceived helplessness against stress showing time effects for control and intervention groups.

**FIGURE 6:**
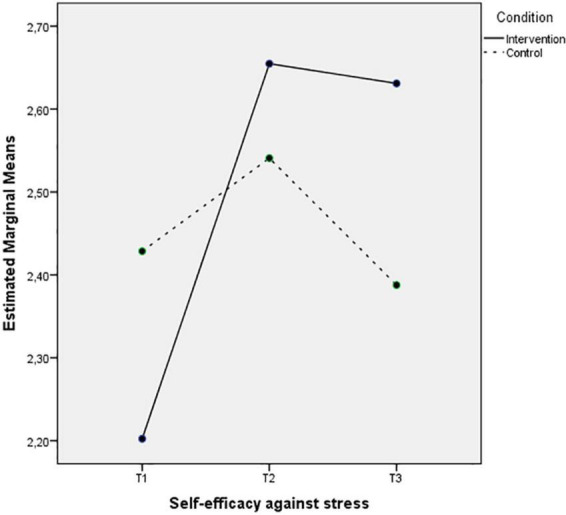
Mean of self-efficacy against stress showing time effects for control and intervention groups.

On the other hand, Figure 5 depict a decrease on the levels of perceived helplessness against stress in Time 2 for the intervention group, half of which remains 3 months after the intervention, while the levels of the control group seem to increase in the second time-point and bounce back to the previous levels in Time 3. The pattern of the interaction between Time and Condition for the self-efficacy against stress is different (see Figure 6). The intervention group levels clearly increase in Time 2 and the same levels remain in Time 3. However, the self-efficacy levels of the control group have slight changes between the three time-points.

**FIGURE 7:**
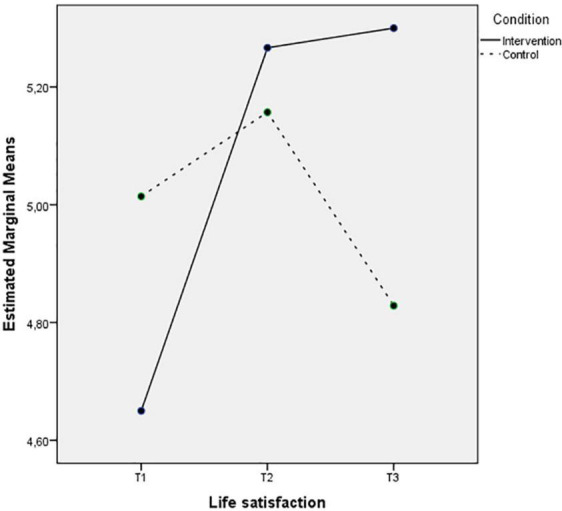
Mean of life satisfaction showing time effects for control and intervention groups.

**FIGURE 8:**
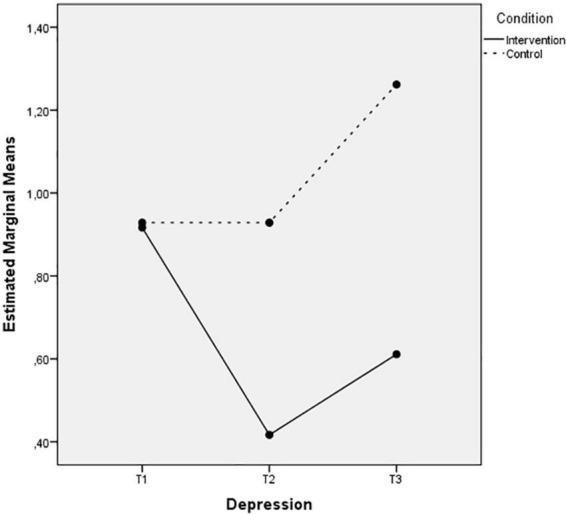
Mean of depression showing time effects for control and intervention groups.

The last two Figures 7, 8 also depict an increase of the life satisfaction levels and a decrease of the depression levels in Time 2 that remain 3 months after the intervention, while the control group levels of these wellbeing indices remain the same or even decrease during the three measurements.

### *Post-hocs* for interactions

To interpret the interactions between Time and Condition more precisely, we ran paired *t*-tests for each group between pairs of the three time-points and independent samples *t*-test between the groups for the three time-points. A Bonferroni correction was applied to the significance level (0.05/6 = 0.008).

The results indicate statistically significant differences only for the intervention group (see Table 3) regarding (a) SIEM between T1–T2 [*t*(11) = −7.745, *p* < 0.001, Cohen’s *D* = −4,67] and T1–T3 [*t*(11) = −3.915, *p* = 0.002, Cohen’s *D* = −2,36], (b) SIDM between T1–T2 [*t*(11) = 7.487, *p* < 0.001, Cohen’s *D* = 4,51] and T1–T3 [*t*(11) = 5.500, *p* < 0.001, Cohen’s *D* = 3,32], (c) self-efficacy against stress between T1–T2 [t(11) = −3.681, *p* = 0.004, Cohen’s *D* = −2,21], and (d) life satisfaction between T1–T2 [*t*(11) = −3.300, *p* = 0.008, Cohen’s *D* = −1,99] and T1–T3 [*t*(11) = −3.341, *p* = 0.007, Cohen’s *D* = −2.01]. More specifically (see Table 4), the participants in the intervention group reported significantly higher levels of SIEM (T1: *M* = 1.15, *SD* = 0.71; T2: *M* = 2.56, *SD* = 0.72; T3: *M* = 2.29, *SD* = 1.09) and life satisfaction (T1: M = 2.20, SD = 0.57; T2: *M* = 2.65, SD = 0.44; T3: *M* = 2.63, SD = 0.42), and lower levels of SIDM that remain significant 3 months after the intervention (T1: *M* = 2.85, *SD* = 0.76; T2: *M* = 1.19, *SD* = 0.53; T3: *M* = 1.42, *SD* = 0.73). Moreover, the participants in the intervention group reported higher levels of self-efficacy against stress after the intervention (T1: *M* = 2.20, *SD* = 0.57; T2: *M* = 2.65, *SD* = 0.44). The small sample size in each group and the Bonferroni correction didn’t result to other significant results, even though the levels of several variables tend to change in different time-points. No statistically significant differences were found in the control group.

**TABLE 3:** Paired-samples *t*-test coefficients for the mean comparison of pairs between the three time-points as a function of condition.

Pairs		*t*	*df*	*p*
**Condition: Intervention**				
SIEM	T1-T2	–7.745	11	0.000
	T1–T3	–3.915	11	0.002
	T2–T3	1.458	11	0.173
SIDM	T1–T2	7.487	11	0.000
	T1–T3	5.500	11	0.000
	T2–T3	–1.217	11	0.249
Positive emotions	T1–T2	–2.479	11	0.031
	T1–T3	–2.309	11	0.041
	T2–T3	0.000	11	1.00
Negative emotions	T1–T2	2.420	11	0.034
	T1–T3	1.581	11	0.142
	T2–T3	–1.773	11	0.104
Perceived helplessness	T1–T2	2.244	11	0.046
against stress	T1–T3	0.921	11	0.377
	T2–T3	–1.797	11	0.100
Self-efficacy	T1–T2	–3.681	11	0.004
against stress	T1–T3	–2.913	11	0.014
	T2–T3	0.297	11	0.772
Life satisfaction	T1–T2	–3.300	11	0.008
	T1–T3	–3.341	11	0.007
	T2–T3	–0.248	11	0.809
Depression	T1–T2	2.514	11	0.029
	T1–T3	1.328	11	0.211
	T2–T3	–1.103	11	0.294
**Condition: Control**				
SIEM	T1–T2	–1.883	13	0.082
	T1–T3	–1.749	13	0.104
	T2–T3	–1.011	13	0.330
SIDM	T1–T2	0.540	13	0.598
	T1–T3	0.667	13	0.516
	T2–T3	0.418	13	0.682
Positive emotions	T1–T2	–0.641	13	0.533
	T1–T3	0.704	13	0.494
	T2–T3	0.987	13	0.342
Negative emotions	T1–T2	0.147	13	0.885
	T1–T3	–1.179	13	0.260
	T2–T3	–1.058	13	0.309
Perceived helplessness	T1–T2	–2.253	13	0.042
against stress	T1–T3	0.175	13	0.864
	T2–T3	1.177	13	0.260
Self-efficacy	T1–T2	–1.282	13	0.222
against stress	T1–T3	0.446	13	0.663
	T2–T3	1.112	13	0.286
Life satisfaction	T1–T2	–1.278	13	0.224
	T1–T3	1.139	13	0.275
	T2–T3	2.880	13	0.013
Depression	T1–T2	0.000	13	1.00
	T1–T3	–3.017	13	0.010
	T2–T3	–2.248	13	0.043

**TABLE 4:** Mean (standard deviation) of each variable as a function of time and condition (*N* = 26).

Variable	Time	Intervention group	Control group
SIEM	T1	1.15 (0.71)	1.93 (0.99)
	T2	2.56 (0.72)	2.03 (0.95)
	T3	2.29 (1.09)	2.21 (0.87)
SIDM	T1	2.85 (0.76)	2.34 (0.86)
	T2	1.19 (0.53)	2.27 (0.85)
	T3	1.42 (0.73)	2.20 (0.99)
Positive emotions	T1	3.21 (0.72)	3.43 (0.81)
	T2	3.69 (0.49)	3.48 (0.73)
	T3	3.69 (0.47)	3.32 (0.87)
Negative emotions	T1	2.83 (0.76)	2.57 (0.83)
	T2	2.25 (0.51)	2.55 (0.80)
	T3	2.42 (0.65)	2.75 (1.05)
Perceived helplessness against stress	T1	2.27 (0.59)	2.10 (0.55)
	T2	1.94 (0.54)	2.23 (0.57)
	T3	2.11 (0.65)	2.08 (0.65)
Self-efficacy against stress	T1	2.20 (0.57)	2.42 (0.75)
	T2	2.65 (0.44)	2.54 (0.67)
	T3	2.63 (0.42)	2.39 (0.80)
Life satisfaction	T1	4.65 (1.18)	5.01 (1.13)
	T2	5.27 (0.83)	5.15 (0.92)
	T3	5.30 (0.84)	4.82 (1.12)
Depression	T1	0.92 (0.71)	0.93 (0.68)
	T2	0.42 (0.40)	0.93 (0.76)
	T3	0.61 (0.66)	1.26 (0.85)

Additionally, statistical significant differences were found between the two conditions in the second time-point regarding the SIDM levels [*t*(22.157) = −3.929, *p* = 0.001 Cohen’s *D* = 1,52; see Table 5]. Based on Table 4, the participants of the intervention group reported significantly lower levels of SIDM comparing to the control group (intervention: *M* = 1.19, *SD* = 0.53; control: *M* = 2.27, *SD* = 0.85). No other significant differences emerged, since the variable levels in the two groups were not equal in the first time-point.

**TABLE 5:** Independent samples *t*-test coefficients for the mean comparison of control and intervention groups as a function of time.

Variables based on Time	*t*	*df*	*p*
SIEM T1	–2.267	24	0.033
SIEM T2	1.563	24	0.131
SIEM T3	0.202	24	0.842
SIDM T1	1.596	24	0.124
SIDM T2	–3.929	22.157	0.001
SIDM T3	–2.246	24	0.034
Positive emotions T1	–0.726	24	0.475
Positive emotions T2	0.827	24	0.417
Positive emotions T3	1.364	20.476	0.187
Negative emotions T1	0.829	24	0.415
Negative emotions T2	–1.126	24	0.271
Negative emotions T3	–0.954	24	0.349
Perceived helplessness against stress T1	0.762	24	0.453
Perceived helplessness against stress T2	–1.340	24	0.193
Perceived helplessness against stress T3	0.100	24	0.921
Self-efficacy against stress T1	–0.859	24	0.399
Self-efficacy against stress T2	0.505	24	0.618
Self-efficacy against stress T3	0.950	24	0.351
Life satisfaction T1	–0.803	24	0.430
Life satisfaction T2	0.315	24	0.756
Life satisfaction T3	1.196	24	0.244
Depression T1	–0.043	24	0.966
Depression T2	–2.079	24	0.048
Depression T3	–2.149	24	0.042

## Discussion

The results of the present study support the notion that stress mindset can be changed to improve stress responses by influencing the way stress is psychologically experienced and behaviorally approached; thus, the findings support that a change in stress mindset affects individual levels of psychological symptoms and wellbeing (Crum et al., 2013, 2017).

More specifically, answering the first and second research question, the “ReStress Mindset” intervention led participants to a statistically significant increase in SIE mindset that remained 3 months after the completion of the program, and a statistically significant decrease in SID mindset that also remained in the follow up measurement. These results are confirming previous research findings regarding the shift of the university students’ stress mindset to a more SIE mindset and a less SID mindset following the completion of the “ReStress Mindset” intervention in the midst of COVID-19 pandemic (Karampas et al., 2022).

Furthermore, the results answered the third research question indicating a statistically significant increase in the levels of life satisfaction that remained significant 3 months after the intervention. Moreover, the participants in the intervention group reported statistically significant higher levels of self-efficacy against stress after the intervention. These findings are in line with previous research indicating that SIE mindset is positively correlated with increased life satisfaction, and positive ways of perceiving stress (self-efficacy when confronting stress) (Crum et al., 2013; Karampas et al., 2020).

The findings also answered the fourth research question, since it was found that the levels of positive emotions increased in the intervention group, while the levels of negative emotions, depression, and perceived helplessness against stress decreased after the intervention and remained unchanged 3 months later, while no statistically significant differences were found in the control group. These findings are consistent with previous research indicating that SIE mindset predicted positive affect, decreased depressive symptoms, and lower negative feelings (perceived helplessness when confronting stress and negative emotions) (Crum et al., 2013; Kilby and Sherman, 2016; Karampas et al., 2020).

The findings of this study have significant implications for university students during the COVID-19 pandemic, since they are facing severe lifestyle and mental health disruptions, which have profound ramifications for their wellbeing (Cao et al., 2020; Li et al., 2020; Liu et al., 2020; McGinty et al., 2020; Tang et al., 2020; Giuntella et al., 2021). To begin with, shifting to a more SIE mindset and away from a more SID mindset is critical since stress mindset affects both the extent to which stress is psychologically experienced and the way stress is behaviorally approached, two variables that are crucial in determining health and performance outcomes under stress (Crum et al., 2013; Jamieson et al., 2013). Moreover, satisfaction with life, defined as the judgmental component of subjective wellbeing, is thought to be a significant predictor of mental and physical health (Diener et al., 1985; Diener, 2012). Furthermore, self-efficacy is regarded as a powerful motivational, cognitive, and affective determinant of university student behavior, with a significant impact on involvement, effort, persistence, self-regulation, and achievement (Schunk and Pajares, 2010; Honicke and Broadbent, 2016; Ritchie, 2016; Zumbrunn et al., 2020). Self-efficacy is an important variable in stress management because of these characteristics (Bandura et al., 2003; Şahin and Çetin, 2017; Lannin et al., 2019), and it is considered as a protective factor against the impact of day-to-day stressors at university (Freire et al., 2018; Schönfeld et al., 2019).

To end up with, the findings of the present study support the notion that a vast repertoire of coping strategies, and flexibility in their selection, may be the best methods to effectively cope with stress and protect university students from decreased wellbeing, during the COVID-19 pandemic (Rogowska et al., 2021a).

### Limitations and recommendations for future research

The results of this study should be viewed in light of its methodological limitations, which should be addressed in future research. First, the study’s sample was small, with the vast majority of participants being women. Furthermore, the study’s sample only consists of Psychology university students, who are often motivated about the process. In addition, participants volunteered to participate in the survey in response to an online invitation, which may mean that they were actively seeking support in relation to stress. To ensure that the findings are generalizable, future studies should aim to recruit a larger and more diverse sample in terms of gender, age, career, and marital status. Furthermore, participants only completed self-reported scales, which by definition are highly subjective and can be influenced by external factors. In future surveys, interviews could be used to collect qualitative data. Future designs will also benefit from the addition of follow-up measures beyond the 3 months examined in this study to reinforce the findings and investigate the long-term impact of the changes identified following the program’s completion.

In the shadow of COVID-19 pandemic, the findings of this study have important research and clinical implications. To begin with, the material of this program is easily adoptable and implementable by campus-based resources such as health centers, counseling centers, health promotion offices, student affairs staff, and other support services to assist university students in effectively coping with stress and protecting themselves from decreased wellbeing during this period of unprecedented disruption and uncertainty. Moreover, since the material of the “ReStress Mindset” intervention is delivered online, it is easily transferable to other populations and or settings; thus, future research could focus on individuals who are at risk of developing stress-related physical or mental health problems. Finally, considering the effectiveness of the “ReStress Mindset” intervention, mental health practitioners and counseling psychologists may find inspiration for internet-delivered stress mindset interventions during COVID-19 pandemic.

## Conclusion

The “ReStress Mindset” intervention resulted in a statistically significant increase in “stress-is-enhancing” mindset (SIEM), life satisfaction, and self-efficacy against stress, as well as a statistically significant decrease in “stress-is-debilitating” mindset (SIDM), with these effects lasting 3 months after the program’s completion. The findings of this study suggest that university students could benefit from the “ReStress Mindset” intervention in order to cultivate and maintain a positive stress mindset and increase their life satisfaction and self-efficacy against stress, even during the COVID-19 pandemic or any other highly stressful period or crisis.

## Data availability statement

The raw data supporting the conclusions of this article will be made available by the authors, without undue reservation.

## Ethics statement

Ethical review and approval was not required for the study on human participants in accordance with the local legislation and institutional requirements. The patients/participants provided their written informed consent to participate in this study.

## Author contributions

All authors have contributed equally to the development of the study’s idea, data acquisition, data analysis, and writing of the manuscript.
